# Impact of anthro-metabolic indices and gestational weight gain on maternal and neonatal outcomes: a prospective observational study

**DOI:** 10.1590/1806-9282.20231101

**Published:** 2024-03-25

**Authors:** Esra Keles, Leyla Kaya, Neşe Yakşi, Zahide Kaya, Önder Tosun

**Affiliations:** 1 University of Health Sciences, Kartal Lütfi Kırdar City Hospital, Department of Gynecologic Oncology – İstanbul, Turkey.; 2 University of Health Sciences, Zeynep Kamil Women and Children’s Disease Training and Research Hospital, Department of Obstetrics and Gynecology – İstanbul, Turkey.; 3 Amasya University, School of Medicine, Department of Public Health – Amasya, Turkey.; 4 Uskudar State Hospital, Internal Medicine Clinic – İstanbul, Turkey.; 5 University of Health Sciences, Zeynep Kamil Women and Children’s Disease Training and Research Hospital, Department of Urogynecology and Endometriosis – İstanbul, Turkey.

**Keywords:** Waist-to-hip ratio, Body mass index, Cesarean section, Macrosomia, Weight gain

## Abstract

**OBJECTIVE:**

The aim of this study was to examine the relationship of anthro-metabolic indices on maternal and neonatal outcomes.

**METHODS:**

This prospective observational study was conducted on healthy mother–baby pairs between January 1, 2023 and July 1, 2023. Detailed sociodemographic information was collected through an interview with the mother. Clinical, biochemical, obstetric, fetal, and neonatal outcomes were abstracted from hospital medical records. Anthropometric measurements were obtained from the examination of mother–baby pairs.

**RESULTS:**

A total of 336 healthy mothers–children pairs were included. Mothers of newborn ≥4000 g had higher gestational age (p=0.003), body mass index (p=0.003), gestational weight gain (p=0.016), waist circumferences (p=0.002), and hip circumferences (p=0.001). gestational weight gain was associated with the mode of delivery (p=0.023). waist-to-hip ratio (p=0.005), gestational weight gain (p=0.013), and a body shape ındex (p<0.001) were associated with longer length of hospital stay. Age (p<0.001) and inter-pregnancy interval (p=0.004) were higher in pre-pregnancy underweight/obese mothers. Receiver operating characteristic analysis revealed that maternal waist circumferences (AUC: 0.708, p=0.005), maternal weight (AUC: 0.690, p=0.010), and hip circumferences (AUC: 0.680, p=0.015) were sufficient to predict macrosomia (p<0.05).

**CONCLUSION:**

The study demonstrated a significant association between gestational weight gain and cesarean delivery, prolonged hospital stay, and macrosomia. It was also found that maternal body mass index, waist circumferences, and hip circumferences during pregnancy were associated with macrosomia. On the contrary, no significant relationship was found between maternal anthro-metabolic characteristics and maternal–fetal and birth outcomes.

## INTRODUCTION

Overweight and obesity have reached epidemic proportions, causing more than 4 million deaths worldwide annually^1^. World Health Organization (WHO) notes that worlwide prevalence of obesity almost tripled over the past four decades^2^. In the United States, more than 50% of pregnant women are suffering from obesity, while in England, 21.3% of pregnant women are living with obesity^3,4^. Previous research has established that a high prepregnancy body mass index (BMI) and gestational weight gain (GWG) are linked to unfavorable maternal and neonatal outcomes, including gestational diabetes, preeclamsia, cesarean delivery, and fetal macrosomia^5,6^.

Body mass index is a commonly used risk stratification tool in pregnancy. However, a disadvantage of BMI is that it does not differentiate fat and lean mass, or reflect fat distribution^7^. It is assumed that all women with obesity are at equal risk of having a poor pregnancy outcome. However, a study involving 5,628 women with uncomplicated pregnancies found that 47% of women with obesity did not experience any adverse pregnancy outcome, whereas 42% of overweight women did^8^. Consequently, BMI has been questioned because it does not accurately predict which women are at high risk of an obesity-related adverse outcome of pregnancy. Therefore, alternative obesity anthropometric indicies have been developed to modulate the limitations of BMI.

Anthropometry is a simple, reliable, and low-cost method and provides useful information regarding abdominal and genitofemoral adiposity^9^. Identification of the effect of those anthropometric parameters on maternal and neonatal outcomes is important in order to reduce or prevent adverse obstetric and neonatal outcomes, which has many implications for the development of maternal and newborn health. Accumulating evidence implies that the distribution of body fat might be a more precise indicator of individual risk, yet there is a dearth of reliable evidence during pregnancy. With this study, we aimed to examine the relationship of anthro-metabolic indices with maternal and neonatal outcomes.

## METHODS

This prospective observational study was carried out at a tertiary hospital from January 1, 2023 to July 1, 2023. Ethical approval was obtained from the Research Ethics Committee (Approval number: 197/22.12.2021) and adheres to the principles of Declaration of Helsinki. All participants gave written informed consent prior to entering the study.

The study recruited a total of 320 healthy pregnant women who gave birth to normal healthy, full-term, singleton baby, with minimum reading and writing literacy, and were willing to participate in the study after being informed of its purpose and methodology. Pregnant women with multiple pregnancies, fetal chromosomal aneuploidy and/or congenital deformities, stillbirth, birth before 37 weeks, previous cesarean section, pregnant women who were using any drugs that affect blood glucose, history of taking alcohol, smoking, those with a history of psychological or physical illnesses, history of complicated pregnancy, who had an addiction, those with chronic diseases (e.g., diabetes, chronic hypertension, liver or kidney disease, cardiovascular disease, and thyroid dysfunction), pregnant women with maternal and fetal complications (e.g., preterm birth, prelabor rupture of membranes, preeclampsia, gestational hypertension, gestational diabetes mellitus, oligohydramnios, polyhydramnios, intrahepatic cholelithiasis, placenta previa, and intrauterine growth restriction) were excluded from the study.

Body weight and height of the expectant mother were measured with a digital weight and height scale. Each participant was required to remove shoes, stand upright with arms loosely to the side, and be positioned in the Frankfurt plane, with equal weight distribution. Body weight and height were measured to the nearest 0.1 kg and 0.001 m, respectively.

Hip circumstance (HC), waist circumstance (WC), mid-upper arm circumstance (MUAC), and neonatal head circumference were measured with a non-stretch tape. A standard technique was followed for accurate anthropometric measurements. After emptying the bladder, the participants removed their clothing and footwear, then stood upright with arms hanging loosely at the sides. WC measurement was made at midpoint between the lowest rib and iliac crest during expiration. HC measurement was made at the widest part of the gluteus region over the greater trochanters. Mid-upper arm circumference (MUAC) was measured halfway between the acromion and the olecranon fossa on the non-dominant arm.

Body mass index was categorized according to WHO^10^. GWG is categorized according to the 2009 Institute of Medicine (IOM) recommendations^11^.

Body mass index formula^12^: weight (kg)/[height (m)]^2^.

A body shape index (ABSI) formula^13^: ABSI=WC(m)/[BMI ^2/3^ × Height (m)^1/2^].

The body round index (BRI) formula^13^: BRI=365.2 - 365.5 × ^√^(1 - (((WC/2π)^2^)/[(0.5 × height)]^2^))

Waist-to-hip ratio (WHR) formula: WHR =WC/HC

Birth weight and lengths of infants were measured within 1 h of birth using standardized procedures. We weighed the neonates naked using a digital weighing scale, in a supine position, to the nearest 0.001 kg. An infant meter was used to measure a baby’s length. On the board, the body was placed with the legs fully extended, and moderate pressure was applied to the knees. After positioning the head, measurements were taken to 0.001 m.

The date of the last menstrual period was taken to determine gestational age, and confirmation was made with first trimester sonographic crown-rump length measurement. Systolic and diastolic blood pressures were taken during delivery time. The blood pressure values were recorded as millimeters wof mercury (mmHg). Detailed sociodemographic information was collected through an interview with the mother. Clinical, biochemical, obstetric, fetal, and neonatal outcomes were abstracted for each patient from patients’ hospital medical records. Anthropometric measurements were obtained from the examination of mother–baby pairs. According to The American College of Obstetricians and Gynaecologists, fetal macrosomia was defined as fetal birth weight greater than 4000 g or 4500 g^14^. In the present study, an infant’s birth weight above >4000 g was defined as fetal macrosomia. APGAR score of 7 points or less was classified as abnormal, and an APGAR score above 7 was classified as normal^15^.

### Statistical analysis

The data collected in the study were transferred to the Epi info 7.2 program and analyzed. Descriptive data are presented in the form of mean, standard deviation, minimum, maximum, number, and percentage values. Whether the distributions of continuous variables were normal or not was controlled with the Kolmogorov-Smirnov test. For comparison between two variables which do not conform to the normal distribution, the Mann-Whitney U-test and Kruskal-Wallis test were employed. A p-value of less than 0.05 was deemed to be indicative of statistical significance.

## RESULTS

A total of 336 mothers–children pairs were included in our study. The mean of the maternal age was 27.53±5.07 years, pre-pregnancy BMI 24.13±4.15 kg/m^2^, and GWG 14.92±6.83 kg. There were 16 women (4.76%) who gave birth to newborns with macrosomia >4000 g, and 8 women (2.38%) had newborns <2500 g. There were 126 (29.7%) women with pre-pregnancy BMI≥25 kg/m^2^, of whom 35 (10.8%) were obese (BMI≥30 kg/m^2^). The percentage of women with excessive GWG was 50.2% (169/336).

In the comparison of maternal characteristics and neonatal birth weight, mothers of newborn ≥4000 g had higher gestational age (p=0.003), BMI (p=0.003), GWG (p=0.016), WC (p=0.002), and HC (p=0.001). Birth weight did not significantly differ according to WHR, MUAC, ABSI, and BRI. Neonatal birth weight was associated with maternal fasting blood glucose (p=0.004), but not with systolic and diastolic blood pressure or hemoglobin value (p>0.05) (Table 1).

**Table 1:** Comparison of neonatal birth weight categories with maternal anthropometric features

Variables	Birth weights (g)			p-value
	Low birth weight (<2500 g)	Normal birth weight (>2500 g)	Macrosomia (>4000 g)	
Maternal age (years)	30.00 (21.00–37.00)	27.00 (18.00–43.00)	27.00 (22.00–38.00)	0.571^¥^
Gestational age (weeks)	37.20 (37.00–41.00)	39.60 (37.00–42.00)	40.65 (38.00–42.00)	**0.003** ^¥^
Maternal BMI (kg/m^2^)	25.87 (18.37–37.46)	29.39 (21.10–42.76)	31.24 (23.57–46.06)	**0.039** ^¥^
Maternal weight gain during pregnancy (kg)	10.50 (2.00–20.00)	15.00 (-9.00–40.00)	18.50 (10.00–31.00)	**0.016** ^¥^
Maternal waist circumference (cm)	101.00 (90.00–118.00)	110.00 (90.00–129.00)	116.00 (106.00–130.00)	**0.002** ^¥^
Maternal hip circumference (cm)	100.00 (90.00–116.00)	112.00 (90.00–132.00)	116.00 (100.0–128.00)	**0.001** ^¥^
A Body shape index (ABSI) (m11/6 kg2/3)	0.92 (0.82–1.01)	0.91 (0.73–1.07)	0.89 (0.73–1.03)	0.867^¥^
Body roundness ındex (BRI)	6.55 (4.26–9.23)	7.45 (4.33–12.46)	7.76 (6.28–9.55)	0.175^¥^
Maternal glucose level	72.10 (67.30–78.90)	83.80 (51.00–185.00)	79.60 (66.40–116.70)	**0.004** ^¥^

Body mass index, BMI. Categorical variables are shown as n (column %), and continous variables are shown as median (min–max).

^¥^Kruskal-Wallis test. Statistically significant values are denoted in bold.

Gestational weight gain was associated with the mode of delivery (p=0.023). The mean diastolic blood pressure was 66.49±7.29 in the spontaneous vaginal delivery (SVD) group, whereas it was 68.92±7.09 in the cesarian section (CS) group (p=0.017) (Table 2).

**Table 2:** Comparison of mode of delivery with maternal anthropometric

Variables	Mode of delivery		p-value
	Spontaneous vaginal delivery	Cesarian section	
Maternal Age (years)	27.00 (18.00–43.00)	27.00 (18.00–40.00)	0.670^¥^
Gestational age (weeks)	39.60 (37.00–42.00)	40.00 (37.00–42.00)	0.060^¥^
Maternal weight gain during pregnancy (kg)	15.00 (-9.00–40.00)	17.00 (-3.00–40.00)	**0.023** ^¥^
Maternal waist circumference (cm)	110.00 (90.00–128.00)	110.00 (94.00–130.00)	0.478^¥^
Maternal hip circumference (cm)	112.00 (90.00–132.00)	110.00 (96.00–128.00)	0.131^¥^
Waist/hip ratio (WHR)	0.98 (0.86–1.16)	1.00 (0.89–1.19)	0.057^¥^
Mid-upper arm circumference (MUAC) (cm)	28.00 (21.00–36.00)	27.00 (24.00–34.00)	0.096^¥^
A body shape index (ABSI) (m11/6 kg2/3)	0.91 (0.73–1.07)	0.90 (0.73–1.00)	0.086^¥^
Body roundness index (BRI)	7.44 (4.26–12.46)	7.35 (5.05–9.98)	0.881^¥^
Systolic blood pressure (mmHg)	110.00 (90.00–130.00)	110.00 (100.00–130.00)	0.580^¥^
Diastolic blood pressure (mmHg)	70.00 (50.00–80.00)	70.00 (50.00–80.00)	**0.017** ^¥^

Body mass index, BMI. Categorical variables are shown as n (column %), continous variables are shown as median (min–max).

^¥^Mann-Whitney U-test. Statistically significant values are denoted in bold.

It was found that maternal anthropometric measurements were not associated with 1-min APGAR score (p>0.05). GWG (p=0.013), WHR (p=0.005), and ABSI (p<0.001) were associated with longer length of hospital stay. APGAR score ≤7 at 1 min was significantly higher in younger mothers (p=0.036) (Table 3).

**Table 3:** Comparison of APGAR score at 1-min and length of hospital stay with maternal anthropometric features

Variables	APGAR score at 1 min		p-value	Length of hospital stay		p-value
	Normal (≥7)	Abnormal (<7)		Normal (≤2)	Abnormal (<2)	
Maternal age (years)	27 (18–43)	24 (19–30)	**0.036** ^¥^	27 (18–43)	27 (18–39)	0.732^¥^
Gestational age (weeks)	39.60 (37.00–42.00)	40.30 (37.00–41.50)	0.150^¥^	39.60 (37.00–42.00)	39.40 (37.00–42.00)	0.479^¥^
Maternal weight gain during pregnancy (kg)	15.00 (-9.00–40.00)	17.00 (1.00–30.00)	0.733^¥^	14.00 (-5.00–38.00)	16.00 (-9.00–40.00)	**0.013** ^¥^
Maternal waist circumference (cm)	110 (90–130)	110 (96–129)	0.976^¥^	110 (90–129)	110 (90–130)	0.155^¥^
Maternal hip circumference (cm)	112 (90–132)	110 (98–120)	0.414^¥^	112 (90–132)	112 (96–128)	0.448^¥^
Waist/hip ratio (WHR)	0.98 (0.86–1.19)	1.00 (0.92–1.13)	0.520^¥^	1.00 (0.87–1.19)	0.98 (0.86–1.13)	**0.005** ^¥^
A body shape index (ABSI) (m11/6 kg2/3)	0.91 (0.73–1.07)	0.92 (0.84–1.00)	0.586^¥^	0.91 (0.73–1.07)	0.89 (0.73–1.00)	**<0.001** ^¥^
Body roundness index (BRI)	7.43 (4.26–12.46)	7.57 (5.63–9.53)	0.825^¥^	7.56 (4.26–12.46)	7.34 (4.33–9.98)	0.195^¥^

Body mass index, BMI. Categorical variables are shown as n (column %), continous variables are shown as median (min–max).

^¥^Mann-Whitney U-Test. Statistically significant values are denoted in bold.

Age (p<0.001) and inter-pregnancy interval (p=0.004) were higher in pre-pregnancy underweight/obese mothers. Neonatal birth height was found to be shorter in pre-pregnancy underweight mothers (p=0.046).

Receiver operating characteristic (ROC) analysis revealed that maternal WC (AUC: 0.708, p=0.005), maternal weight (AUC: 0.690, p=0.010), and HC (AUC: 0.680, p=0.015) were sufficient to predict macrosomia (p<0.05).

## DISCUSSION

The study demonstrated a significant association between GWG and cesarean delivery, prolonged hospital stay, and macrosomia. It was also found that maternal BMI, WC, and HC measurements during pregnancy were associated with macrosomia. On the contrary, no significant relationship was found between maternal pre-pregnancy weight, BRI, ABSI, MUAC, WHR, and maternal–fetal and birth outcomes.

It is well established that excessive GWG is a risk factor for macrosomia, regardless of pre-pregnancy BMI^16^. The results of our study corroborate the findings of other studies, including a recent meta-analysis involving 1,309,136 women^17^. The researchers reported that high GWG was associated with macrosomia and cesarean delivery. Similarly, a multicenter study also found associations between GWG and adverse pregnancy outcomes, including macrosomia, shoulder dystocia, cesarean birth, and neonatal hypoglycemia^18^. Another prospective cohort study reported that excessive GWG played a crucial role in macrosomia prediction^19^. These findings suggest that GWG is critical in maternal and neonatal outcomes. Thus, effective public health interventions are necessary in order to prevent excess gestational weight gain^20^.

Nguyen et al., found that women delivering macrosomic babies had higher WC compared with controls^21^. Likewise, a large follow-up study by Li et al., suggested that GWG and high WC but not WHR were risk factors for macrosomia^22^. In contrast, a Mendelian randomization analysis of Geng et al., did not find any causal relationship between maternal WC, WHR, and birth weight. However, they noted that genetically predisposed to higher HC was linked to increased birth weight^23^. In our data, WC and HC were associated with macrosomia. According to these data, we can infer that WC and HC measured in the third trimester may be the useful predictors for macrosomia.

Alternative anthropometric measures that standardize BMI, such as ABSI and BRI, have been developed to reflect the health status. In the study of Özler et al., examining the anthropometric indices in the first trimester pregnant women demonstrated that BRI, but not ABSI, may be a reliable predictor for fetal macrosomia in obese pregnant women^24^. Conversely, the present study found no association between these two indices and neonatal and birth outcomes. A possible explanation for this might be that our study was conducted on healthy women in their third trimester of pregnancy.

This study has several limitations. The present study did not incorporate pre-pregnancy anthropometric characteristics. To address this shortcoming, future studies would benefit from longitudinal data extending from pre-pregnancy period to the postpartum period. It is important to note that, in contrast with previous studies, this study was unique in that participants were measured by a trained health professional, and data were collected prospectively, allowing for accurate data. Unlike studies that focus primarily on the association between anthropometric characteristics and cardiometabolic diseases in a non-pregnant population, this study provides a comprehensive assessment of a variety of anthro-metabolic indices within a specific population. Considering increasing maternal obesity rates as well as the lack of evidence relating to health outcomes in mother–infant dyads, research is of paramount importance. The findings established in this study will guide health care providers and policy makers in developing early intervention strategies.

## CONCLUSION

This large, diverse cohort with prospectively collected data showed that maternal BMI, GWG, WC, and HC during pregnancy are important factors in determining clinical and fetal outcomes. Promoting optimal weight gain during pregnancy may reduce adverse maternal and neonatal complications.

## Data Availability

The dataset used and analyzed in the study is available from the corresponding author on reasonable request.
